# Warming Alters Non-Structural Carbohydrate Dynamics and Source–Sink Regulation in Two Temperate Tree Species

**DOI:** 10.3390/plants15172598

**Published:** 2026-08-26

**Authors:** Jingyao Ren, Jingge Hu, Zhaoxing Li, Lei Jin, Kai Huang, Yilin Wang, Yunze Leng, Jiang Liu, Xiufen Li

**Affiliations:** 1 College of Agronomy, Shenyang Agricultural University, Shenyang 110866, China; renjingyao2024@syau.edu.cn (J.R.); lll122624@163.com (Z.L.); yilinyilin345@gmail.com (Y.W.); 15841522757@163.com (Y.L.); 2 Fushun Meteorological Services, Fushun 113006, China; m13029264199@163.com; 3 Dalian Meteorological Bureau, Dalian 116001, China; jl1207900231@163.com; 4 Key Laboratory of Forest Ecology and Management, Institute of Applied Ecology, Chinese Academy of Sciences, Shenyang 110016, China; huangkai@iae.ac.cn; 5 Qingyuan Forest CERN, National Observation and Research Station, Shenyang 110016, China; 6 National Field Observation and Research Station (Liaoning Qingyuan) for Forest Ecosystem, Fushun 113300, China

**Keywords:** field warming, photosynthetic physiology, non-structural carbohydrates, carbon allocation, species-specific responses

## Abstract

Understanding how trees coordinate carbon acquisition and allocation under warming is essential for predicting forest–carbon dynamics; however, whether warming induces common or species-specific source–sink regulation strategies remains unresolved. We conducted a field warming experiment (+2 °C and +4 °C above ambient) to investigate photosynthetic responses, organ-specific non-structural carbohydrate (NSC) dynamics, and carbon allocation strategies in two temperate tree species, *Fraxinus mandschurica* and *Juglans mandshurica*. Moderate warming (+2 °C) initially enhanced carbon assimilation in both species, whereas severe warming (+4 °C) progressively inhibited photosynthetic performance during the later growing season. In *F. mandschurica*, +2 °C warming promoted carbon storage by increasing starch accumulation across leaves, stems, and roots, with starch concentrations 2.5–38.0% higher than those in control plants from July to September. These enhanced NSC reserves were positively associated with photosynthetic performance and survival, indicating an effective source-driven carbon storage strategy. In contrast, *J. mandshurica* exhibited a distinct carbon allocation pattern under warming, characterized by preferential soluble sugar accumulation in leaves and reduced carbon investment in storage tissues. Specifically, leaf soluble sugar-to-starch ratios increased by 6.0–162.3%, whereas root soluble sugar content decreased by up to 55.7% under warming, suggesting a shift toward short-term carbon regulation rather than long-term storage. Structural equation modeling further demonstrated that photosynthetic performance and leaf NSC pools were central regulators of warming responses, but their effects on downstream carbon allocation differed between species. Our results reveal that warming does not induce a uniform carbon response among temperate trees; instead, species-specific source–sink regulation strategies determine carbon resilience under elevated temperatures. These findings improve understanding of how coexisting tree species may diverge in their adaptive capacity under future climate warming.

## 1. Introduction

Increased greenhouse gas emissions from human activities have profoundly altered the global climate system, leading to a marked rise in surface temperature [1]. Current estimates indicate global temperatures have already increased by approximately 1.25 °C due to anthropogenic influence, with projections suggesting a likely exceedance of the 1.5 °C threshold within the coming decade [2]. Notably, this warming exhibits significant spatial heterogeneity, with land surfaces warming more rapidly than oceans [3]. However, the mechanisms underlying how long-lived trees maintain carbon balance under warming conditions remain poorly understood. In particular, whether trees can compensate for warming-induced increases in carbon demand through adjustments in carbon acquisition and allocation remains a critical uncertainty for predicting forest–carbon dynamics under future climate scenarios [4]. This uncertainty arises because tree carbon balance depends not only on carbon acquisition through photosynthesis but also on the coordination between carbon sources and sinks across different organs.

As the dominant structural components and primary carbon reservoirs of forests, trees are central to ecosystem carbon cycling [5]. Their physiological responses to rising temperatures are therefore critical for predicting future forest carbon sequestration potential, ecosystem stability, and community dynamics. The fitness of trees under climatic stress ultimately depends on their carbon balance that the dynamic equilibrium between carbon acquisition, allocation, and utilization [6]. How trees adjust their carbon income (photosynthesis) and carbon allocation and storage (non-structural carbons, NSCs) strategies is the key to understanding their adaptability and vulnerability in terms of physiology [7].

In perennial trees, carbon balance is regulated through dynamic interactions between carbon sources and sinks. Leaves act as major source organs by assimilating atmospheric CO_2_, whereas stems, roots, and developing tissues function as sinks that consume or store assimilated carbon. Under environmental stress, the balance between carbon supply and sink demand determines whether newly assimilated carbon is invested in growth, stored as NSCs, or mobilized for maintenance metabolism. Therefore, understanding warming responses requires an integrated assessment of source activity and sink regulation rather than isolated evaluation of photosynthetic performance.

Photosynthesis, the primary pathway for carbon entry into plants, is highly sensitive to temperature [8]. Elevated temperatures can disrupt the biochemical and biophysical foundations of photosynthesis, including enzyme activity, membrane stability, and stomatal regulation [9]. However, severe or prolonged heat stress can induce stomatal closure, limiting CO_2_ uptake while concurrently increasing respiratory carbon loss, which may lead to a negative carbon balance [10]. Nevertheless, photosynthetic performance alone cannot fully represent the carbon status of trees, because assimilated carbon must subsequently be allocated among competing sinks, including growth, respiration, reproduction, and storage.

Warming may alter the process of photosynthetic carbon acquisition, thereby affecting the source–sink relationship of NSCs, and ultimately driving the redistribution of carbon among organs and components. Carbon balance, achieved through the interplay of photosynthesis, respiration, and growth, is vital for maintaining metabolic processes and responding to environmental stressors [11]. The carbon fixed through photosynthesis is allocated to growth, metabolism, and storage. NSCs, primarily comprising soluble sugars (SS) and starch (ST), serve as crucial mobile carbon reserves and metabolic buffers [12,13,14]. They support essential functions including respiration during non-photosynthetic periods, spring reactivation, osmotic adjustment, and recovery from abiotic stresses [11]. Allocation patterns reflect strategic adaptation to environmental conditions; for instance, preferential allocation to roots may enhance resource foraging under stress [15,16]. Despite increasing recognition of the importance of NSCs in plant responses to climate change, current understanding remains incomplete. Previous studies have mainly focused on changes in total NSC concentrations or individual organs, whereas how warming alters coordinated carbon allocation among source organs and sink organs remains poorly understood. Recent evidence suggests that NSC responses are highly organ-specific, with carbohydrate dynamics in stems, branches, and roots not necessarily reflecting whole-plant carbon status [17]. Addressing these knowledge gaps is essential for understanding how trees regulate carbon balance and maintain growth under future warming scenarios.

To address these knowledge gaps, we selected two dominant temperate deciduous tree species, *F. mandschurica* and *J. mandshurica*, as model species. Both species are important components of temperate forests in Northeast China, but they differ in ecological strategies and resource-use characteristics. *F. mandschurica* generally exhibits relatively rapid growth and higher resource acquisition capacity, whereas *J. mandshurica* tends to invest more in structural tissues and long-term carbon storage. Therefore, comparing these two co-occurring species provides an opportunity to determine whether warming responses are regulated by common physiological mechanisms or species-specific carbon allocation strategies. Specifically, we examine the coordination between carbon acquisition and allocation across tissues and assess how warming modifies source–sink relationships and carbon balance at the whole-plant level.

## 2. Results

### 2.1. The Influence of Field Warming on the Survival Rate and Relative Growth Rate

**Figure 1:**
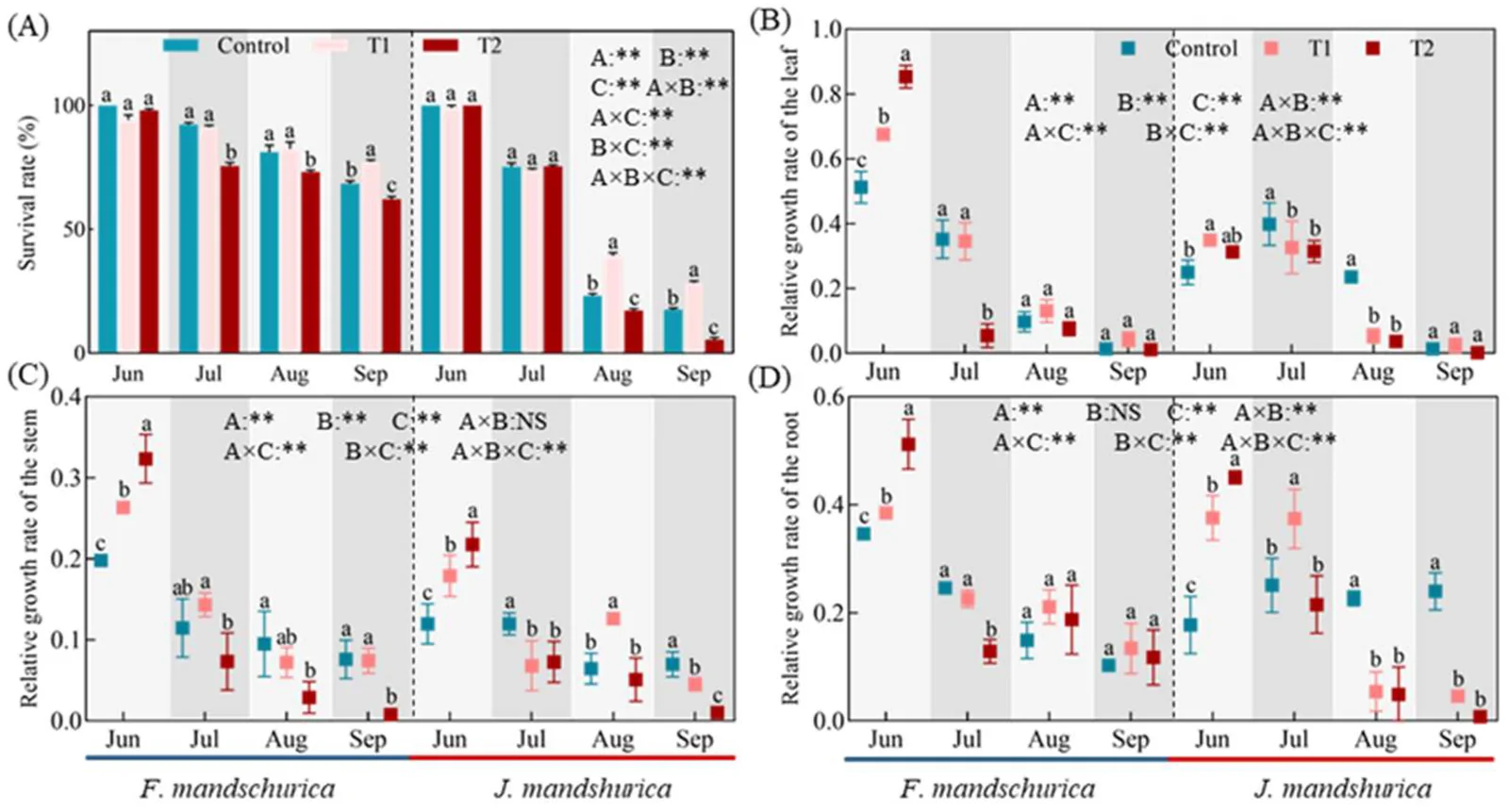
Effects of warming on survival rate and relative growth rate of *F. mandschurica* and *J. mandshurica* seedlings. (**A**) Survival rate; (**B**) relative growth rate of leaves; (**C**) relative growth rate of stems; and (**D**) relative growth rate of roots. Different lowercase letters indicate significant differences among different treatments (*p* < 0.05). The uppercase letters indicate experimental factors (A = variety, B = treatment, C = sampling time). The error bars indicate the standard errors of the means. ** significant difference at the 0.01 probability level; NS, no significant difference.

As indicated in Table S1, survival rates and relative growth rates (RGR) differed significantly between *F. mandschurica* and *J. mandshurica*, with warming effects strongly dependent on intensity (Figure 1). Moderate warming (T1) increased survival in both species, whereas severe warming (T2) significantly reduced survival, indicating that excessive warming imposed physiological constraints on seedling performance. In June, warming generally promoted tissue RGR; however, in July, T2 significantly decreased stem and leaf RGR in both *F. mandschurica* (36.0% and 84.4%) and *J. mandshurica* (16.4% and 34.9%). From August to September, warming had limited effects on *F. mandschurica*, whereas it significantly reduced root and stem RGR in *J. mandshurica*, suggesting greater growth resilience of *F. mandschurica* and stronger belowground growth sensitivity of *J. mandshurica* under prolonged warming.

### 2.2. The Influence of Field Warming on the Photosynthetic Characteristics

**Figure 2:**
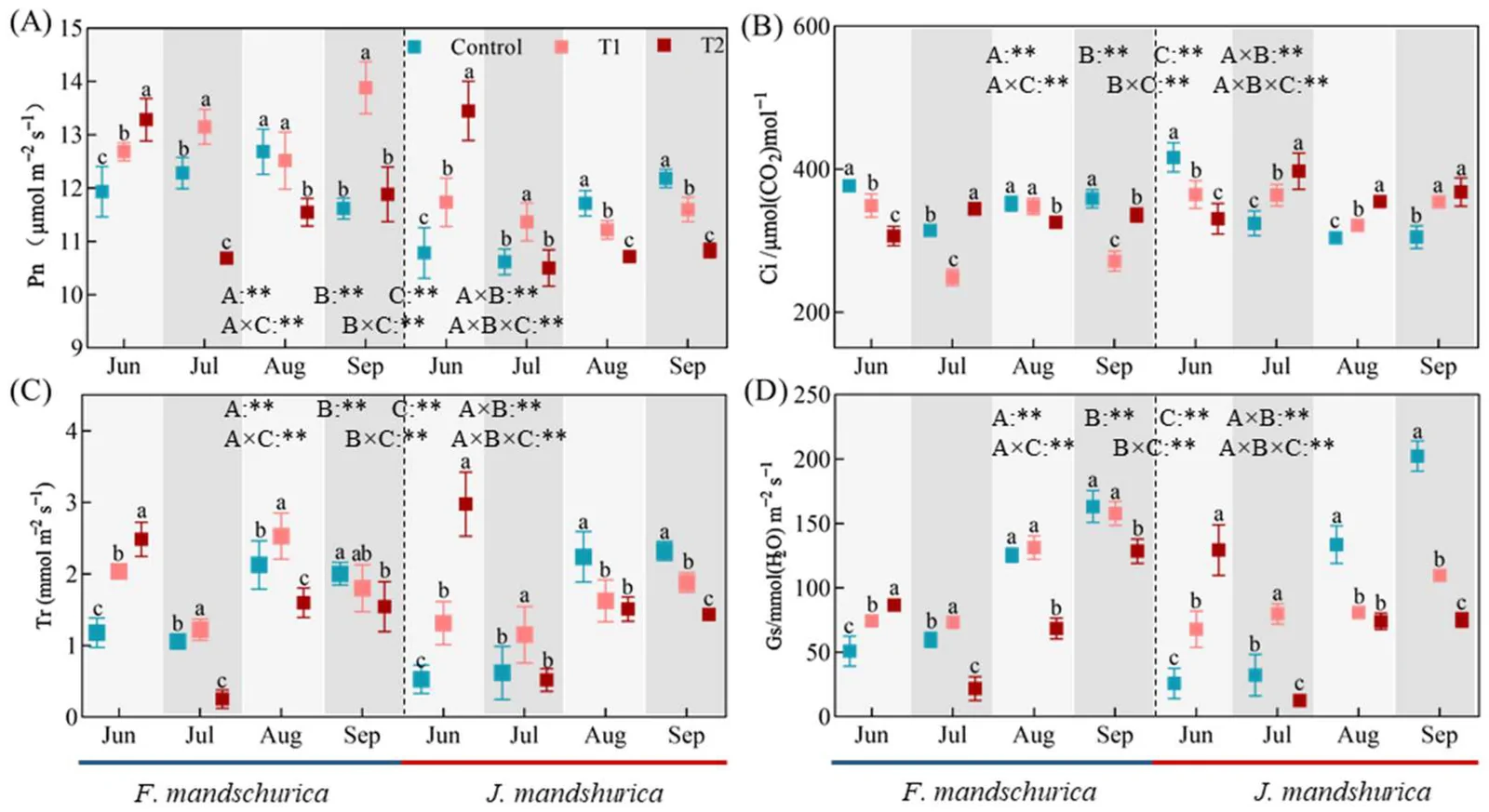
Photosynthetic characteristics of *F. mandschurica* and *J. mandshurica* seedlings. (**A**) Net photosynthetic rate, Pn. (**B**) Intercellular CO_2_ concentration, Ci. (**C**) Transpiration rate, Tr. (**D**) Stomatal conductance, Gs. Different lowercase letters indicate significant differences among different treatments (*p* < 0.05). The uppercase letters indicate experimental factors (A = variety, B = treatment, C = sampling time). The error bars indicate the standard errors of the means. ** significant difference at the 0.01 probability level.

As illustrated in Figure 2 and Table S1, warming intensity strongly regulated photosynthetic responses in both species. Moderate warming (T1) enhanced photosynthetic performance, particularly in *F. mandschurica*, with Pn increasing by 7.0% in July and 19.5% in September compared with the control treatment. In contrast, severe warming (T2) consistently suppressed Pn, Tr, and Gs in both species, especially during July and August, indicating that excessive warming constrained carbon assimilation. The contrasting patterns of Ci further suggested species-specific photosynthetic regulation: warming generally reduced Ci in *F. mandschurica*, implying enhanced CO_2_ assimilation capacity, whereas increased Ci in *J. mandshurica* from July to September under warming suggested possible limitations in CO_2_ utilization and photosynthetic acclimation.

### 2.3. The Influence of Field Warming on the Chlorophyll Fluorescence Parameters

**Figure 3:**
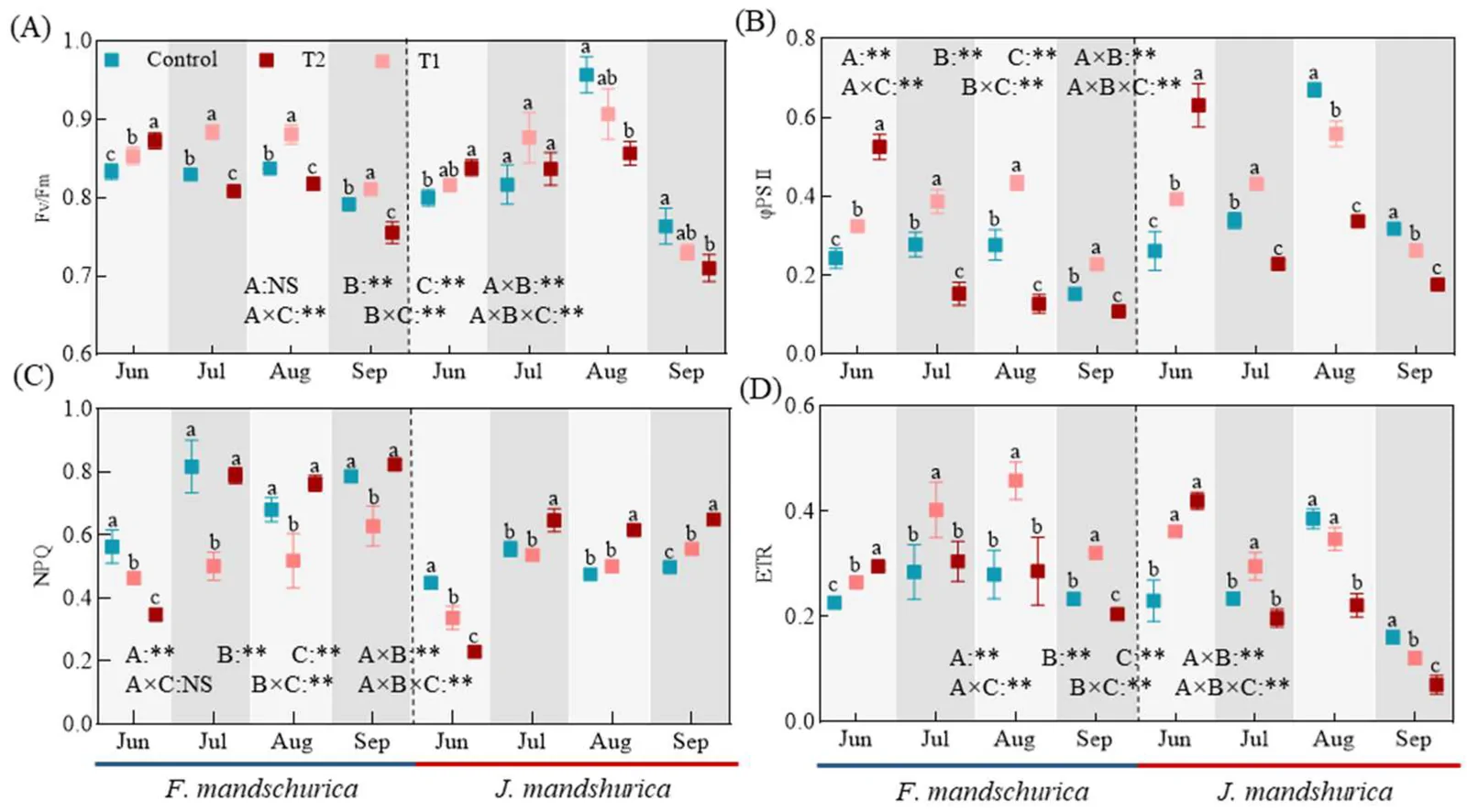
Chlorophyll fluorescence parameters of *F. mandschurica* and *J. mandshurica* seedlings. (**A**) Maximum quantum efficiency of PSII photochemistry, Fv/Fm. (**B**) Effective quantum yield of PSII photochemistry, ΦPSII. (**C**) Non-photochemical quenching, NPQ. (**D**) Electron transport rate, ETR. Different lowercase letters indicate significant differences among different treatments (*p* < 0.05). The uppercase indicate experimental factors (A = variety, B = treatment, C = sampling time). The error bars indicate the standard errors of the means. ** significant difference at the 0.01 probability level; NS, no significant difference.

Chlorophyll fluorescence analysis demonstrated distinct thermal adaptation strategies in *F. mandschurica* and *J. mandshurica* (Figure 3 and Table S1). Moderate warming initially enhanced photochemical efficiency in June, with T2 increasing Fv/Fm by 4.7% and 4.8%, ΦPSII by 116% and 141%, and ETR by 30% and 82% in *F. mandschurica* and *J. mandshurica*, respectively, while reducing NPQ by 38.3% and 48.7%. However, prolonged exposure to severe warming reversed this positive response, with T2 causing progressive declines in Fv/Fm, ΦPSII, and ETR and reaching the lowest values in September. In contrast, T1 maintained relatively higher photochemical activity throughout the growing season, suggesting that moderate warming promoted photosynthetic acclimation, whereas excessive warming impaired PSII function and limited photochemical energy conversion in both species.

### 2.4. The Influence of Field Warming on the NSCs Content and SS/ST Ratio

**Figure 4:**
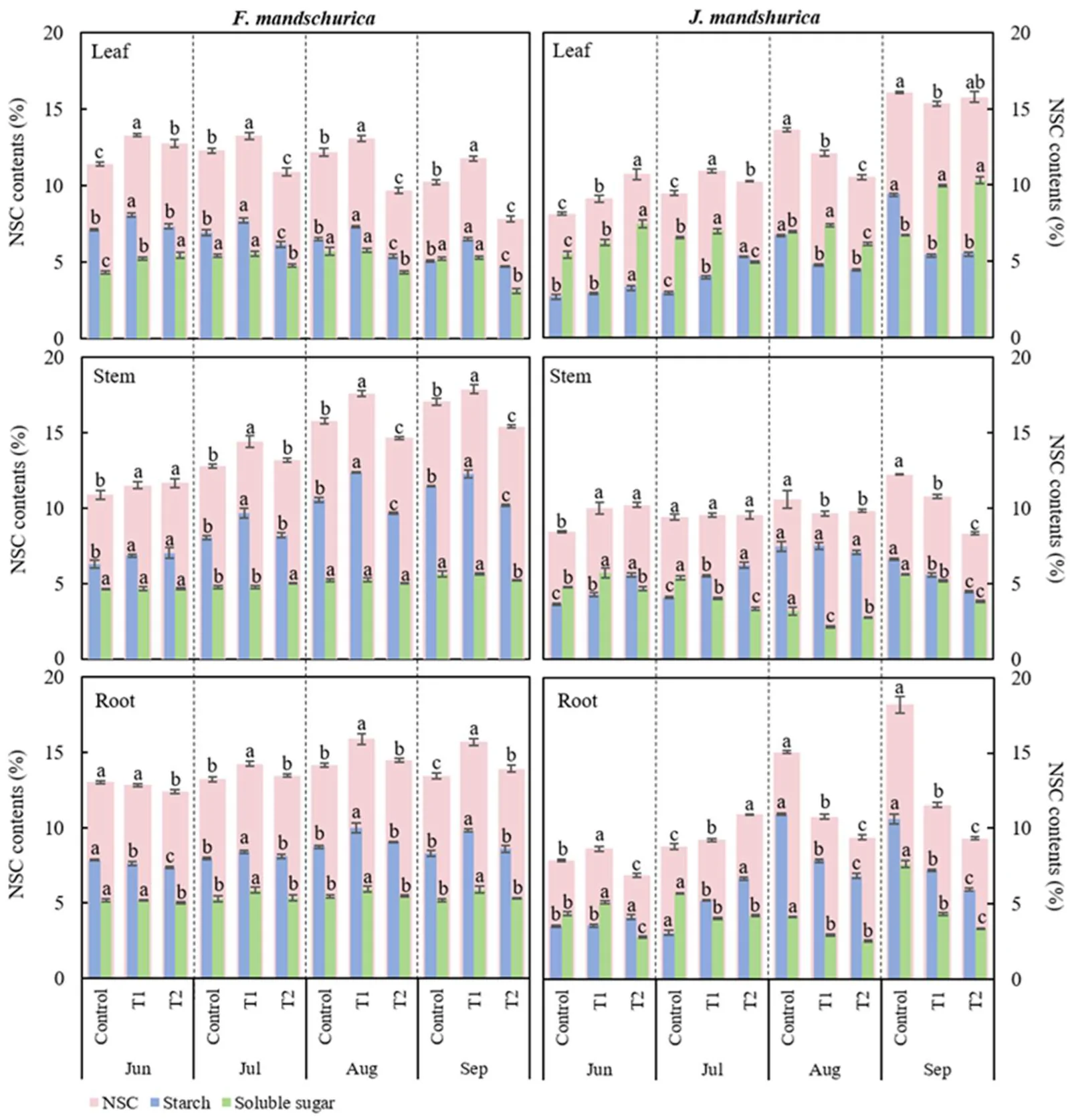
Changes in the NSCs contents of *F. mandschurica* and *J. mandshurica* seedlings. Different lowercase letters indicate significant differences among different treatments (*p* < 0.05).

The dynamics of non-structural carbohydrates (NSCs) exhibited distinct tissue- and species-specific responses to warming (Table S1 and Figure 4). In *F. mandschurica*, SS concentrations remained relatively stable across tissues, with only a slight decrease in leaves under warming in September. In contrast, *J. mandshurica* showed increased leaf SS accumulation but reduced SS levels in stems and roots under warming, with root SS reaching the lowest value under T2 in September (55.7% lower than the control). These contrasting patterns suggest that the two species adopted different strategies for soluble carbon allocation under warming. Starch dynamics further highlighted species-specific carbon storage responses. In *F. mandschurica*, T1 promoted starch accumulation across tissues, with ST concentrations remaining 2.5–38.0% higher than those under control and T2 treatments from July to September, indicating enhanced carbon storage capacity under moderate warming. However, *J. mandshurica* exhibited an initial increase in ST during June–July followed by a temperature-dependent decline from August to September, suggesting that prolonged warming shifted carbon allocation away from starch storage and potentially increased carbon utilization demands.

**Figure 5:**
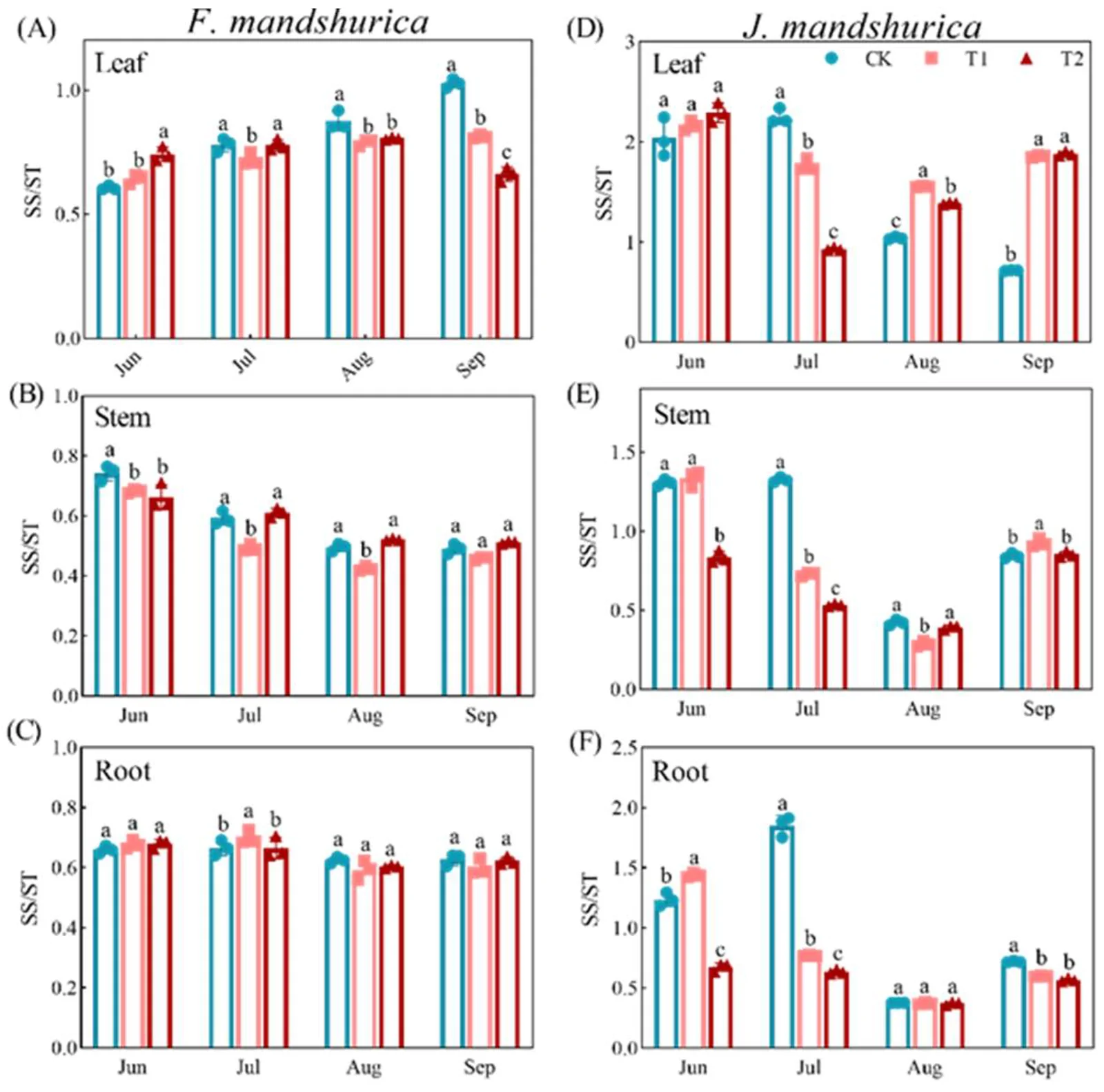
Changes in the SS/ST ratio in leaves, stems, and roots of *F. mandschurica* (**A**–**C**) and *J. mandshurica* (**D**–**F**) seedlings. Different lowercase letters indicate significant differences among different treatments (*p* < 0.05).

In *F. mandschurica*, warming had limited effects on the SS/ST ratio, with leaf values generally decreasing from July to September (0.26–35.6% lower than the control) and only a transient increase occurring in June. Similarly, stem and root SS/ST ratios remained relatively stable, with only a slight increase (4.9%) in roots under T1 in July (Figure 5). In contrast, *J. mandshurica* showed stronger shifts in carbon allocation, characterized by increased soluble sugar proportion in leaves but reduced SS/ST ratios in stems and roots. Leaf SS/ST ratios increased by 6.0–162.3% under warming, whereas stem and root ratios declined markedly, with reductions of 45.0–59.8% in stems and 58.4–65.6% in roots under July warming treatments (Figure 5). These divergent patterns suggest that *F. mandschurica* maintained a relatively stable balance between soluble sugars and starch, while *J. mandshurica* preferentially retained soluble sugars in leaves and reduced carbohydrate availability in belowground tissues, reflecting distinct carbon allocation strategies under warming.

### 2.5. The Influence of Field Warming on the NSCs Distribution and Variability

**Figure 6:**
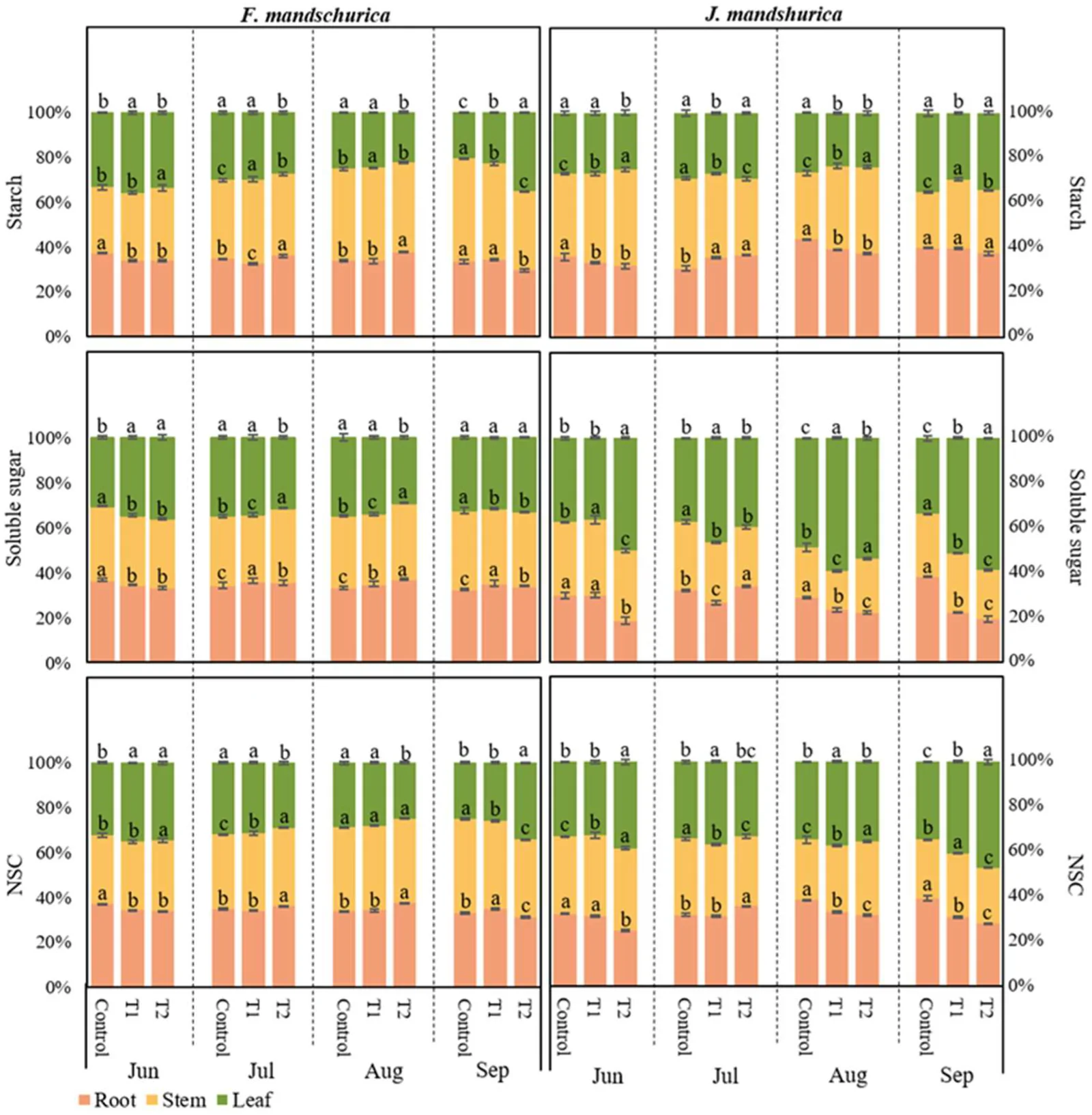
Changes in the NSCs distribution of *F. mandschurica* and *J. mandshurica* seedlings. Different lowercase letters indicate significant differences among different treatments (*p* < 0.05).

We further examined tissue-specific NSC allocation patterns under different warming treatments (Figure 6). Warming induced contrasting carbohydrate allocation strategies between the two species. In *F. mandschurica*, warming generally increased NSC accumulation in leaves and roots but reduced stem sugar content, indicating a relatively balanced carbon allocation pattern among tissues. In contrast, *J. mandshurica* exhibited a stronger shift toward aboveground carbon retention, with leaf sugar proportion increasing by 6.1–74.6% under warming, accompanied by reduced sugar allocation in stems and roots. Temporal analysis further showed that warming initially decreased leaf NSC content in *F. mandschurica* from June to July but enhanced leaf NSC accumulation by September, whereas *J. mandshurica* maintained consistently higher leaf NSC levels, particularly under T1 treatment. These results suggest that *F. mandschurica* may maintain relatively flexible carbon redistribution among tissues, while *J. mandshurica* preferentially retains soluble carbon in leaves under warming conditions.

We subsequently analyzed the variability in NSC content. As illustrated in Figure S1, T1 treatment in *F. mandschurica* increased the levels of ST, SS, and NSC across various tissues, while T2 treatment produced the opposite effect. These differences were most pronounced between July and September. Furthermore, the warming treatments had a more significant impact on leaf NSC content in *F. mandschurica*. In *J. mandshurica*, both T1 and T2 treatments under field warming exhibited generally similar trends in modifying NSC content, with more pronounced effects observed in the roots and leaves.

### 2.6. Correlation Analysis and Correlation Network Construction of F. mandschurica and J. mandshurica Under Field Warming

**Figure 7:**
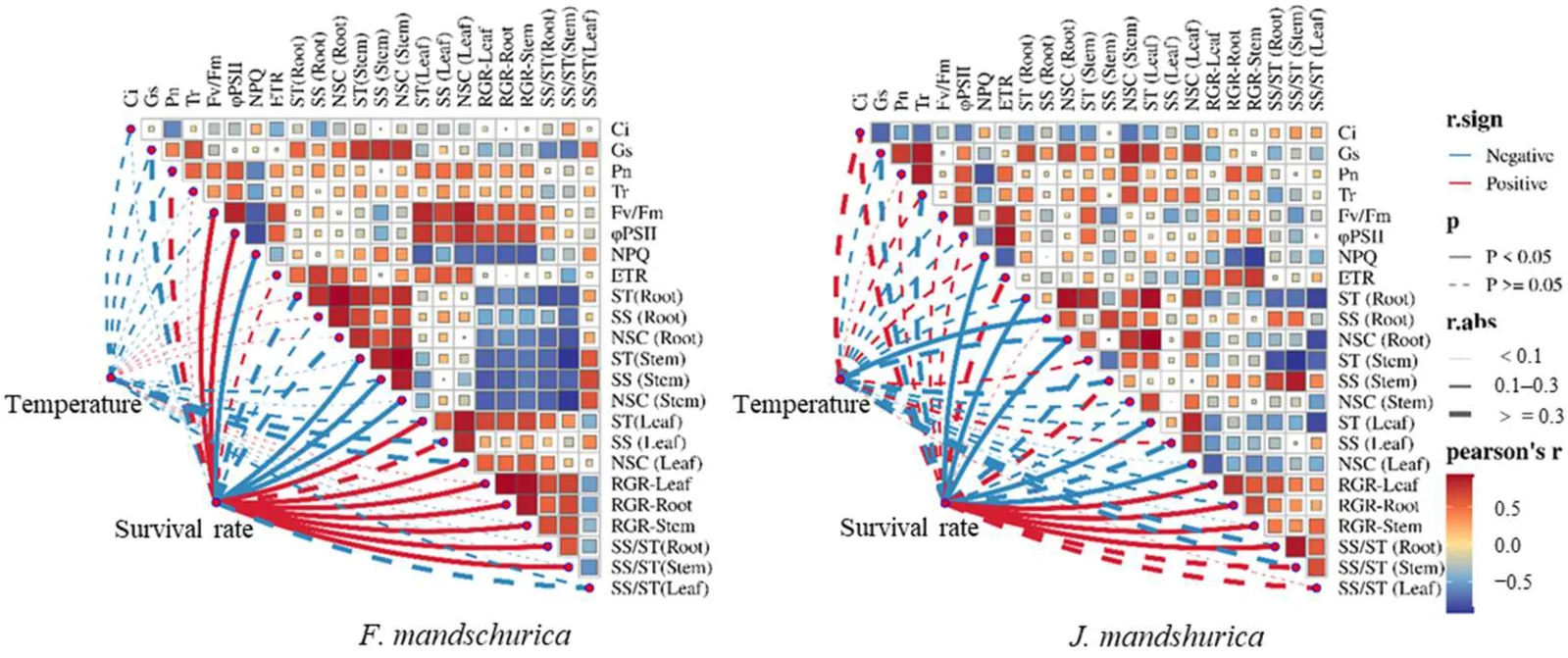
Correlation network heatmap analysis among growth parameters and temperature of *F. mandschurica* and *J. mandshurica*. The lines indicate significant correlations between temperature/survival rate and physiological traits. Red lines represent positive correlations, whereas blue lines represent negative correlations. The thickness of the lines corresponds to the strength of correlations. These relationships represent statistical associations rather than causal effects.

Correlation analysis revealed distinct relationships between physiological traits, carbon status, and growth responses in the two species (Figure 7). In *F. mandschurica*, photochemical efficiency (Fv/Fm and ΦPSII) was positively associated with the growth of leaves, stems, and roots, as well as leaf SS, ST, and NSC contents, indicating that enhanced photosynthetic performance contributed to carbon accumulation and growth maintenance. Leaf ST and NSC contents were also positively correlated with whole-plant growth, whereas stem and root ST and NSC showed negative correlations with RGR, suggesting that carbon stored in different tissues may have contrasting roles in growth regulation. In contrast, *J. mandshurica* exhibited a stronger linkage between aboveground and belowground growth, with leaf RGR positively correlated with root and stem growth. Moreover, stomatal conductance (Gs) was positively correlated with starch accumulation in leaves and roots and NSC content in stems and roots, highlighting the importance of stomatal regulation in carbon acquisition and allocation under warming conditions.

Correlation analysis further revealed contrasting relationships between warming responses, physiological traits, and survival in the two species (Figure 7). In *F. mandschurica*, warming intensity was negatively correlated with photosynthetic performance (Ci, Gs, Pn, and Fv/Fm) and leaf carbohydrate status, including SS, ST, NSC content, and SS/ST ratio, indicating that increased warming constrained carbon acquisition and leaf carbon availability. Conversely, survival rate was positively associated with leaf Pn, Fv/Fm, SS, ST, and NSC contents, suggesting that maintaining photosynthetic capacity and leaf carbon reserves contributed to seedling persistence under warming. In *J. mandshurica*, warming was negatively correlated with several photosynthetic traits (Gs, Fv/Fm, ΦPSII, and ETR) and carbohydrate pools in stems and roots, indicating greater sensitivity of belowground carbon regulation to warming. However, leaf SS content showed positive correlations with both warming intensity and survival rate, suggesting that maintaining soluble carbon in leaves may represent an important adaptive strategy. Furthermore, survival was positively correlated with SS/ST ratios and relative growth rates across leaves, stems, and roots, highlighting the importance of balanced carbon allocation and sustained growth for survival under warming conditions.

A correlation network was constructed to identify key factors based on their correlation patterns. The nodes with red background indicate stronger correlations, signifying essential factors. As shown in Figure S2, RGR-Leaf, RGR-Stem, and NSC (stem) were key factors in *F. mandschurica*. In *J. mandshurica*, ST (root), ST (leaf) and NSC (root) were key factors. It indicated that these key indicators are critical to understanding species-specific carbon economy and adaptive strategies under environmental change.

### 2.7. Integrated Assessment by Structural Equation Modeling

**Figure 8:**
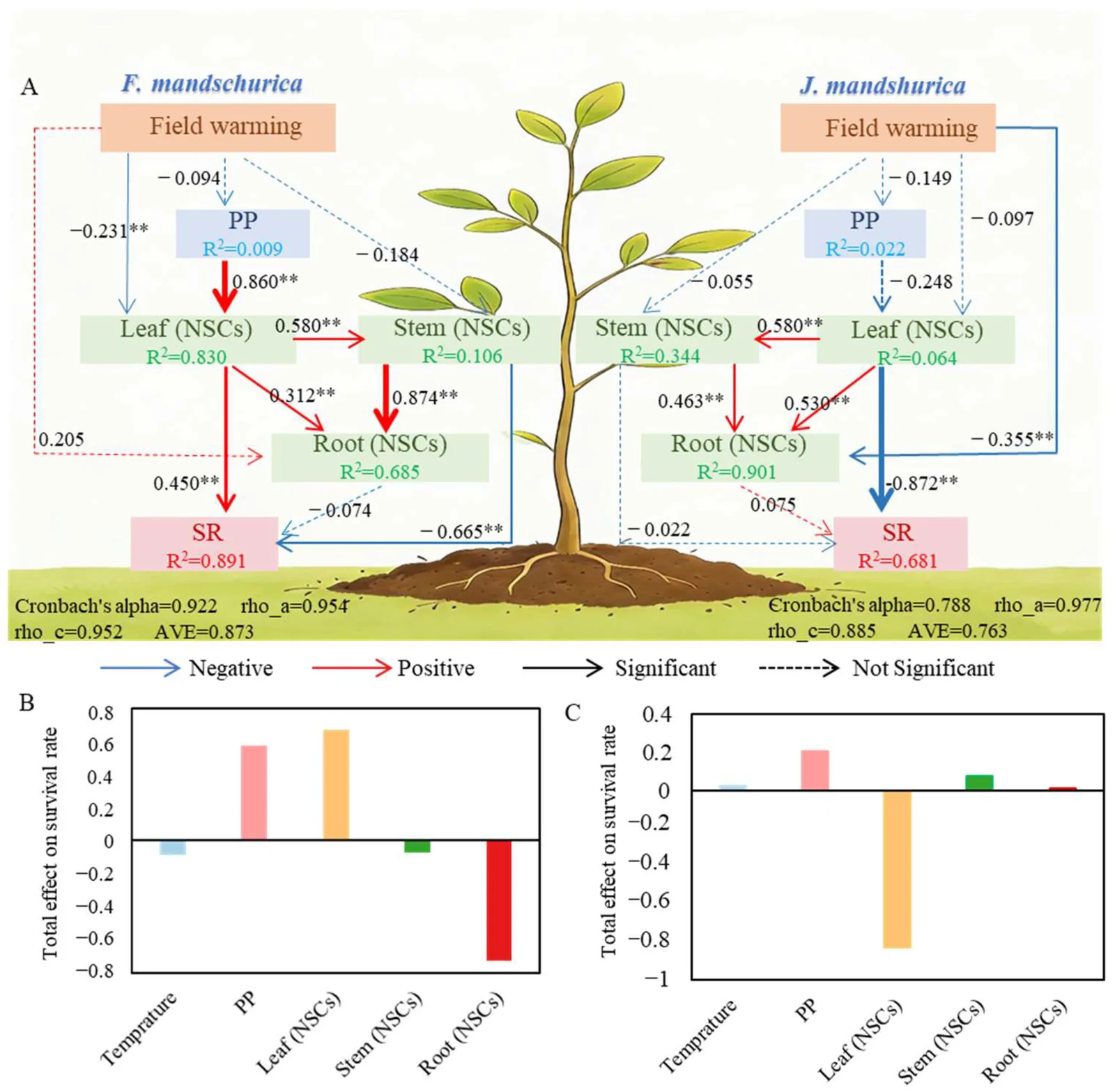
Structural equation modeling based on partial least squares (PLS-SEM) analysis of the effects of temperature on survival rate. (**A**) Path diagram of the PLS-SEM model. (**B**) Standardized effects of temperature on SR evaluation indices of *F. mandschurica*. (**C**) Standardized effects of temperature on SR evaluation indices of *J.* mandshurica. Boxes represent variables included in the model. R^2^ indicates the percentage of variance explained for the corresponding variable. Numbers on the connecting lines represent standardized path coefficients, with their magnitude proportional to the thickness of the lines. ** denote significant effects at the 1% levels. PP, photosynthetic physiology; Leaf (NSCs), soluble sugar, starch and NSC of leaf; Stem (NSCs), soluble sugar, starch and NSC of stem; Root (NSCs), soluble sugar, starch and NSC of root; SR, survival rate.

Structural equation modeling (SEM) revealed that field warming exerted a negative effect on photosynthetic physiology as well as on stem and leaf NSCs in both species (Figure 8A). For *F. mandschurica*, photosynthetic physiology significantly and positively influenced leaf NSC, which in turn significantly promoted NSC accumulation in roots and stems, ultimately enhancing plant survival rate. In contrast, for *J. mandshurica*, leaf NSC was negatively associated with survival rate, despite promoting stem and root NSC content. Path analysis further indicated that in *F. mandschurica*, photosynthetic physiology, leaf NSC, and root NSC collectively had a significant positive effect on overall survival. For *J. mandshurica*, photosynthetic physiology and leaf NSC emerged as the principal factors governing survival rate. Overall, photosynthetic physiology and leaf NSC were identified as key contributors to the integrative responses under warming in both species (Figure 8B,C).

## 3. Discussion

Our results demonstrate that warming responses of temperate tree seedlings were strongly dependent on warming intensity and species identity. Moderate warming (+2 °C) temporarily enhanced photosynthetic performance and carbohydrate accumulation, whereas severe warming (+4 °C) progressively impaired photosynthetic activity and altered carbon allocation patterns. These contrasting responses indicate that warming does not simply increase or decrease plant carbon status, but instead modifies carbon balance through intensity-dependent physiological regulation.

Under heat stress, NSCs undergo rapid hydrolysis, serving as key metabolic buffers that facilitate critical physiological adjustments and maintain cellular homeostasis [18]. ST represents a major energy reserve and carbon pool in plants, and its metabolic dynamics are highly sensitive to abiotic stresses—including drought, osmotic pressure, and notably, temperature fluctuations. Under heat stress, the balance of starch synthesis and degradation is disrupted, directly influencing soluble sugar availability and ultimately affecting the plant’s thermotolerance capacity. SS, particularly sucrose, play crucial roles in energy metabolism, osmotic regulation, and stress signaling, contributing to membrane stabilization and cryoprotection under abiotic stress [19]. ST and sucrose metabolism determine the levels of SS, which are important osmoregulatory substances that alleviate osmotic stress [20].

In this study, NSC responses differed markedly between the two species, suggesting contrasting carbon regulation strategies under warming. In *F. mandschurica*, moderate warming increased starch accumulation across organs (2.5–38.0% higher than control), and leaf NSC was positively associated with photosynthetic performance and survival. This indicates that *F. mandschurica* was able to convert enhanced carbon assimilation into stable storage pools, maintaining carbon availability under warming. This pattern suggests that *F. mandschurica* adopts a storage-oriented strategy, where carbon acquired by source tissues is effectively transported and stored in sink organs. Such coordinated allocation may enhance resilience by buffering future carbon demands during periods of thermal stress. In contrast, *J. mandshurica* exhibited a fundamentally different carbon response.

Although warming increased leaf soluble sugar accumulation, NSC reserves in stems and roots declined, with root soluble sugars decreasing by up to 55.7%. This suggests that warming induced preferential retention of readily available carbon in leaves rather than promoting long-term carbon storage. The accumulation of soluble sugars in leaves may represent an osmotic adjustment strategy or a response to impaired carbon export from source tissues. However, the simultaneous reduction in belowground carbon reserves indicates a potential imbalance between carbon acquisition and sink demand. Although starch generally represents the dominant storage form of NSCs in woody roots, the relatively high SS/ST ratio observed in *J. mandshurica* roots during July suggests that this species may maintain a larger proportion of soluble carbon pools during the active growth period, potentially reflecting rapid carbon turnover and greater metabolic demand.

Photosynthetic responses to warming exhibited a strong dependence on warming intensity and seasonal temperature conditions in this study. Moderate warming (+2 °C) enhanced photosynthetic performance during the early growing season, whereas severe warming (+4 °C) resulted in progressive photosynthetic inhibition as the growing season progressed. This pattern indicates that the temperature increase did not uniformly constrain carbon assimilation, but rather shifted from stimulation to inhibition when thermal conditions exceeded the physiological optimum of the two species. In June, warming treatments increased Pn, Tr, and Gs in both *F. mandschurica* and *J. mandshurica*, suggesting that moderate temperature elevation promoted enzymatic activity and carbon assimilation under relatively favorable thermal conditions. Similar positive responses to moderate warming have been reported in temperate trees, where increased temperature can enhance biochemical processes and extend the period of active carbon gain when other environmental constraints are absent [21]. However, this stimulatory effect weakened under prolonged warming, particularly under the +4 °C treatment. In July and August, when ambient temperatures were highest, both species exhibited reduced Pn and Tr, accompanied by decreased Gs and increased Ci. The combination of reduced stomatal conductance and increased intercellular CO_2_ concentration suggests that the decline in photosynthesis was primarily associated with non-stomatal limitations, including impaired photosynthetic biochemical capacity rather than simple stomatal closure.

The chlorophyll fluorescence results further supported this interpretation. Elevated temperatures impair both PSI and PSII, as well as the dark reactions, leading to suppressed electron transport capacity and reduced oxygen evolution [22]. Within the photosynthetic apparatus, φPSII is among the most heat-sensitive components [23]. Although warming initially increased Fv/Fm, ΦPSII, and ETR in June, prolonged exposure to elevated temperature reduced these parameters, particularly under +4 °C warming. The decline in PSII efficiency indicates that excessive warming impaired photochemical energy conversion, thereby restricting electron transport and downstream carbon fixation. Consequently, reduced photosynthetic carbon input under severe warming likely contributed to the limited NSC accumulation observed in both species, especially in sink organs.

Notably, the two species differed in their ability to maintain photosynthetic function under prolonged warming. In *F. mandschurica*, although photosynthetic parameters declined under severe warming, moderate warming maintained relatively stable photosynthetic performance throughout the growing season. Moreover, the positive correlations between ΦPSII, leaf NSC content, and growth rates suggest that this species was able to effectively translate photosynthetic carbon gain into biomass production and storage. In contrast, *J. mandshurica* showed a more persistent decline in photosynthetic performance under warming, with photosynthetic inhibition continuing until September. This prolonged impairment may have restricted carbon export from source tissues and contributed to the accumulation of soluble sugars in leaves but reduced NSC availability in roots and stems.

Therefore, our results suggest that the impact of warming on tree carbon balance depends not only on the magnitude of temperature increase but also on the duration of exposure and species-specific photosynthetic acclimation capacity. Moderate warming may enhance carbon acquisition when temperatures remain within the optimal range, whereas excessive and prolonged warming disrupts photosynthetic regulation and alters subsequent carbon allocation patterns [24].

Heat stress additionally disrupts enzyme activities, protein structures, and membrane permeability, while downregulating photochemical quenching, carbon assimilation, and sucrose metabolic pathways [25]. In response, plants often redirect carbon resources toward defense and maintenance mechanisms [26]. Photosynthetically fixed carbon is allocated to sink organs in the form of NSC, providing essential energy for metabolic processes in trees [27]. For example, Rehschuh et al. employed ^13^CO_2_ pulse-labeling coupled with ^15^N soil application to track carbon allocation over a 2.5-week period, showing that heat-stressed seedlings rapidly translocate ^13^C to the roots for respiration [28]. In contrast, our results indicated that warming did not significantly increase root NSC content; rather, *J. mandshurica* exhibited a notable reduction, underscoring species-specific strategies in carbon partitioning under thermal stress.

Interestingly, warming effects were strongly seasonal. Early season warming enhanced photosynthetic activity and growth, likely because elevated temperature accelerated metabolic processes under non-limiting thermal conditions. However, during midsummer when ambient temperatures reached their maximum, additional warming exceeded the optimal temperature range and caused persistent photosynthetic inhibition. These results highlight that warming impacts cannot be predicted solely from temperature increase magnitude but depend on the timing of thermal stress. The structural equation modeling results highlight distinct warming-response pathways between *F. mandschurica* and *J. mandshurica*, underscoring species-specific strategies in carbon allocation and survival regulation under thermal stress. In *F. mandschurica*, leaf NSC served as a central hub positively linked to root and stem NSC pools, ultimately supporting higher survival. This pattern is consistent with resource-conservative species that prioritize carbohydrate storage and systemic allocation under stress. Conversely, in *J. mandshurica*, increased leaf NSC was negatively associated with survival despite increased allocation to some sink tissues, suggesting that carbon accumulation in leaves may represent restricted export rather than improved carbon status. Collectively, our findings demonstrate that warming resilience is determined not only by the capacity for carbon acquisition but also by how carbon is partitioned among different organs. *F. mandschurica* maintained carbon balance through coordinated storage and allocation, whereas *J. mandshurica* exhibited a more conservative strategy characterized by leaf carbon retention and reduced belowground investment. These species-specific source–sink regulation patterns provide new insights into how temperate trees may diverge in their adaptive responses under future climate warming.

## 4. Materials and Methods

### 4.1. Study Site and Experimental Design

**Figure 9:**
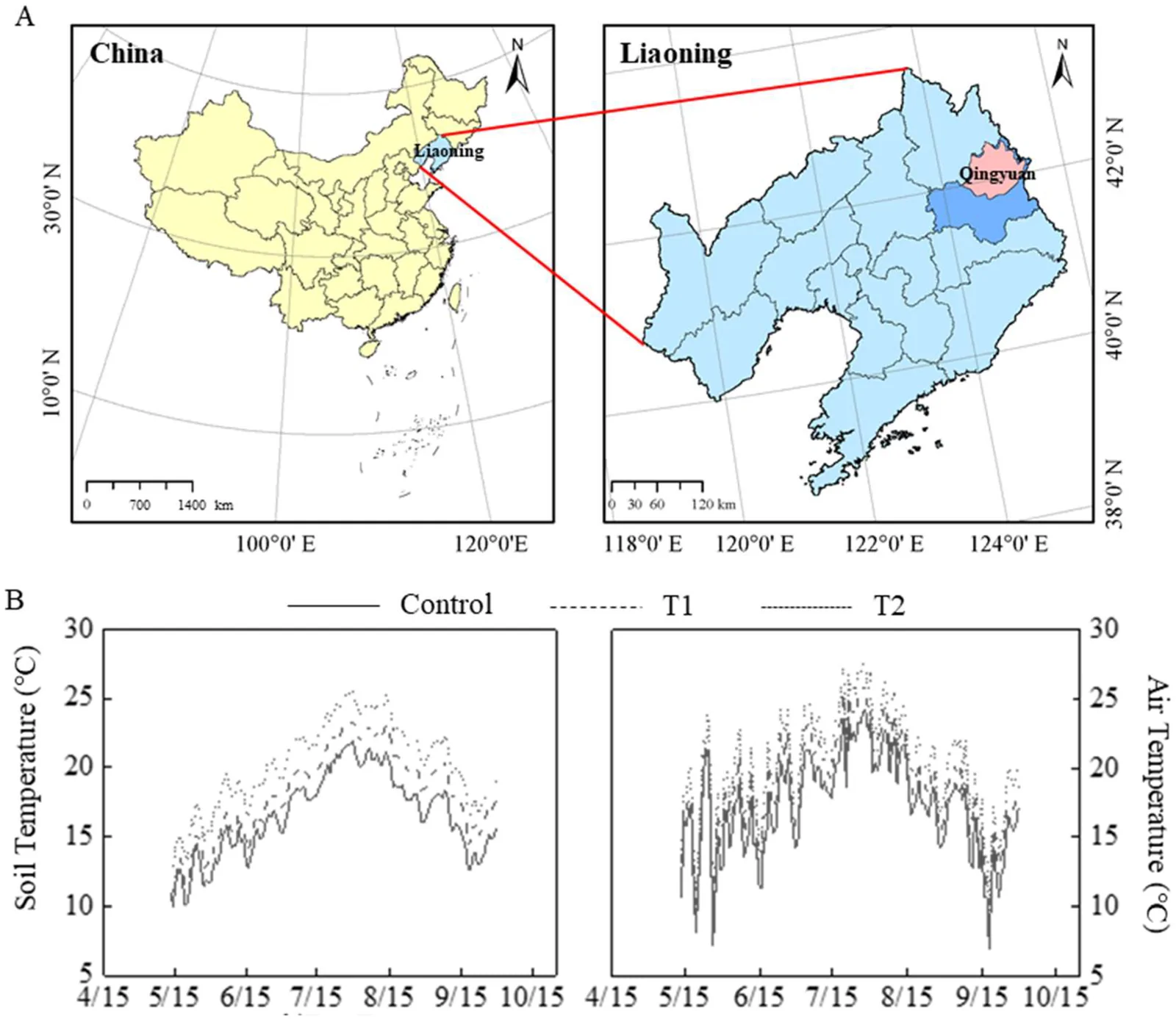
The geographical location of the research site (**A**) and the daily variation curve of mean temperature of the soil and the air of heating gradient (**B**).

The research was carried out at the Qingyuan Forest Station of the Chinese Ecosystem Research Network, National Observation and Research Station, located in Liaoning Province in northeastern China (41°51′ N, 124°54′ E; elevation 500–1100 m above sea level) (Figure 9). One-year-old seedlings of two co-occurring dominant tree species (*F. mandschurica* and *J. mandshurica*) were sourced from Qingyuan Forest CERN, National Observation and Research Station and cultivated in a local nursery. Seedlings of uniform size were then selected and transplanted into experimental warming plots.

The T-FACE system was established in a temperate mixed forest stand. It comprises four main components: infrared heaters, continuous soil temperature monitoring sensors, feedback control units, and a power supply system [29]. The experimental design included three warming treatments: ambient temperature (Control), ambient temperature elevated by 2 °C (T1), and ambient temperature elevated by 4 °C (T2). The experiment employed a randomized block design with three replicates per treatment, resulting in a total of nine plots. Each plot measured 2 m × 1.5 m and was separated from adjacent plots by a buffer zone of at least 5 m. In the warming treatments (T1 and T2), two infrared heaters (220 V; 2000 W for T1 and 4000 W for T2; length: 2.0 m; diameter: 1.8 cm) fitted with reflectors were installed per plot. Control plots were equipped with two non-functional dummy heaters for comparable shading effects. All heaters were suspended 2.0 m above the ground. Soil temperature at a depth of 5 cm was measured every 5 min using PT100 thermocouples, while air temperature at 50 cm above the ground was recorded at 15 min intervals with a Minikin THi sensor (EMS s.r.o., Brno, Czech Republic) (Figure 9). To help maintain stable temperatures, each plot was enclosed with transparent polycarbonate hollow panels (5 mm thick, 1.25 m long), suspended 5 cm above the ground to allow adequate natural ventilation.

Similar-sized one-year-old seedlings of two co-occurring dominant tree species, *F.* and *J. mandshurica*, were obtained from a local nursery and transplanted into the warming plots. Following transplantation, seedlings were allowed to acclimate for 30 days under ambient conditions to minimize potential effects of transplant shock before the initiation of warming treatments. Prior to the warming experiment, all seedlings were examined to ensure comparable growth status, and individuals exhibiting visible signs of transplant stress were excluded.

For each species, seedlings were randomly assigned to three warming treatments, including control (CK), moderate warming (T1), and severe warming (T2), with three independent seedlings per treatment as biological replicates. Biomass and NSC measurements were conducted using three seedlings per species per treatment at each sampling month. Gas exchange and chlorophyll fluorescence measurements were performed on six independent seedlings per species per treatment during each measurement period.

### 4.2. Monitoring of Seedling Growth

The survival rate of the seedlings is calculated using the following formula: Survival rate of seedlings (%) = Number of surviving seedlings/Total number of seedlings. From May to September, the biomass of seedlings across all plots was measured on a monthly basis. Seedling biomass measured immediately after the slow recovery period in May served as the baseline, and the relative growth rate (RGR) was calculated accordingly: RGR = (Ln(w2) − Ln(w1))/(t2 − t1). The w1 represents the initial biomass, w2 represents the final biomass, t1 represents the initial time, and t2 represents the final time.

### 4.3. Measurements of Physiological Indicators

The net photosynthetic rate (Pn), intercellular CO_2_ concentration (Ci), transpiration rate (Tr) and stomatal conductance (Gs) were determined by CIRAS-3 (Hansatech, Pentney, UK). Measurements were performed between 9:00 and 11:00 a.m. on fully expanded, healthy, and intact mature leaves from similar canopy positions. Newly expanded leaves and leaves with visible damage were excluded. The leaf chamber conditions were as follows: a PPFD of 750 μmol∙m^−2^∙s^−1^, relative humidity of 70%, and a CO_2_ concentration of 390 μmol mol^−1^ in the leaf chamber. During measurements, the leaf chamber temperature was maintained close to ambient field temperature to minimize differences between chamber and natural conditions. The leaves were dark-adapted for 30 min, then the chlorophyll fluorescence parameters were performed using FMS-2 (Hansatech, Pentney, UK). For NSC analysis, tissues collected from three seedlings within each treatment were pooled as one sample, and each pooled sample was considered as one biological replicate for statistical analyses. NSCs, including soluble sugars and starch, were quantified using the anthrone–sulfuric acid method following [30]. Approximately 50 mg of dried and finely ground tissue samples were extracted three times with 80% ethanol at 80 °C for 30 min to determine soluble sugar concentrations. The supernatants were collected and reacted with anthrone reagent, and absorbance was measured at 620 nm using a microplate reader. Glucose was used as the standard, and the calibration curve was established using glucose concentrations ranging from 0 to 100 μg mL^−1^.The remaining residues were used for starch determination. Starch was hydrolyzed with 30% perchloric acid at room temperature for 30 min, and the hydrolysates were subjected to the anthrone–sulfuric acid reaction. Absorbance was measured at 620 nm, and starch concentrations were calculated based on the glucose standard curve after applying the conversion factor. NSC in this study was calculated by the sum of total starch contents and soluble sugar contents.

### 4.4. Statistical Analysis

Statistical analyses were performed with Microsoft Excel (Microsoft Corporation, Redmond, WA, USA) and SPSS 22 (SPSS Inc., Chicago, IL, USA), and graphical representations were generated using GraphPad Prism 8 (GraphPad Software, Inc., San Diego, CA, USA). Differences among treatments were assessed by three-way analysis of variance, followed by the least significant difference (LSD) test for post hoc multiple comparisons. Partial least squares structural equation modeling (PLS-SEM) was conducted using SmartPLS 4 software (SmartPLS GmbH, Oststeinbek, Germany).

## 5. Conclusions

In conclusion, experimental warming induced season- and intensity-dependent changes in photosynthetic performance, carbohydrate metabolism, and carbon allocation in *F. mandschurica* and *J. mandshurica*. Moderate warming temporarily enhanced photosynthetic activity and promoted carbon accumulation, whereas prolonged severe warming impaired photosynthetic efficiency and disrupted carbon balance. The two species exhibited contrasting carbon allocation strategies, with *F. mandschurica* maintaining a storage-oriented strategy through enhanced starch accumulation and coordinated organ-level carbon allocation, while *J. mandshurica* preferentially retained soluble sugars in leaves but experienced greater depletion of belowground carbon pools. These differences highlight the importance of species-specific source–sink regulation in determining warming resilience. However, this study was conducted using one-year-old seedlings over a relatively short experimental period, and whether these responses persist in mature trees or under long-term climate warming remains uncertain. Future studies integrating multi-year warming experiments, mature forest ecosystems, and realistic diurnal warming scenarios are needed to better understand long-term tree adaptation mechanisms. Our findings suggest that future silvicultural practices in temperate forests should consider species-specific thermal adaptation strategies. These insights provide a physiological basis for selecting suitable tree species and developing adaptive forest management strategies under future climate warming.

## Data Availability

Data will be made available on request.

## Supplementary Materials

The following supporting information can be downloaded at: https://www.mdpi.com/article/10.3390/plants15172598/s1, Additional File S1: Figure S1: The variability analysis of NSC content of *F. mandschurica* and *J. mandshurica*; Figure S2: Correlation network analysis of growth parameters of *F. mandschurica* and *J. mandshurica*; Additional File S2: Table S1: Analysis of variance of physiology traits of *F. mandschurica* and *J. mandshurica*.
