# Supplementary material for: Warming Alters Non-Structural Carbohydrate Dynamics and Source–Sink Regulation in Two Temperate Tree Species
Source: Plants (Basel). 2026 Aug 26;15(17):2598. doi: 10.3390/plants15172598 (PMC13567195; doi:10.3390/plants15172598)
Supplement: Supplementary file 1 [file plants-15-02598-s001.zip › Additional file S1.pdf]

Article

Warming Alters Non-Structural Carbohydrate Dynamics and Source–Sink Regulation in Two Temperate Tree Species

Jingyao Ren [1,†], Jingge Hu [2,†], Zhaoxing Li [1], Lei Jin [3], Kai Huang [4], Yilin Wang [1], Yunze Leng [1], Jiang Liu [1,*,‡] and Xiufen Li [1,5,6,*,‡]

[1] College of Agronomy, Shenyang Agricultural University, Shenyang 110866, China
[2] Fushun Meteorological Services, Fushun 113006, China
[3] Dalian Meteorological Bureau, Dalian 116001, China
[4] Key Laboratory of Forest Ecology and Management, Institute of Applied Ecology, Chinese Academy of Sciences, Shenyang 110016, China
[5] Qingyuan Forest CERN, National Observation and Research Station, Shenyang 110016, China
[6] National Field Observation and Research Station (Liaoning Qingyuan) for Forest Ecosystem, Fushun 113300, China
* Correspondence: snliujiang@syau.edu.cn (J.L.); lixiufen2019@syau.edu.cn (X.L.)
† These authors contributed equally to this work.
‡ These authors co-supervised this work and are co-corresponding authors.

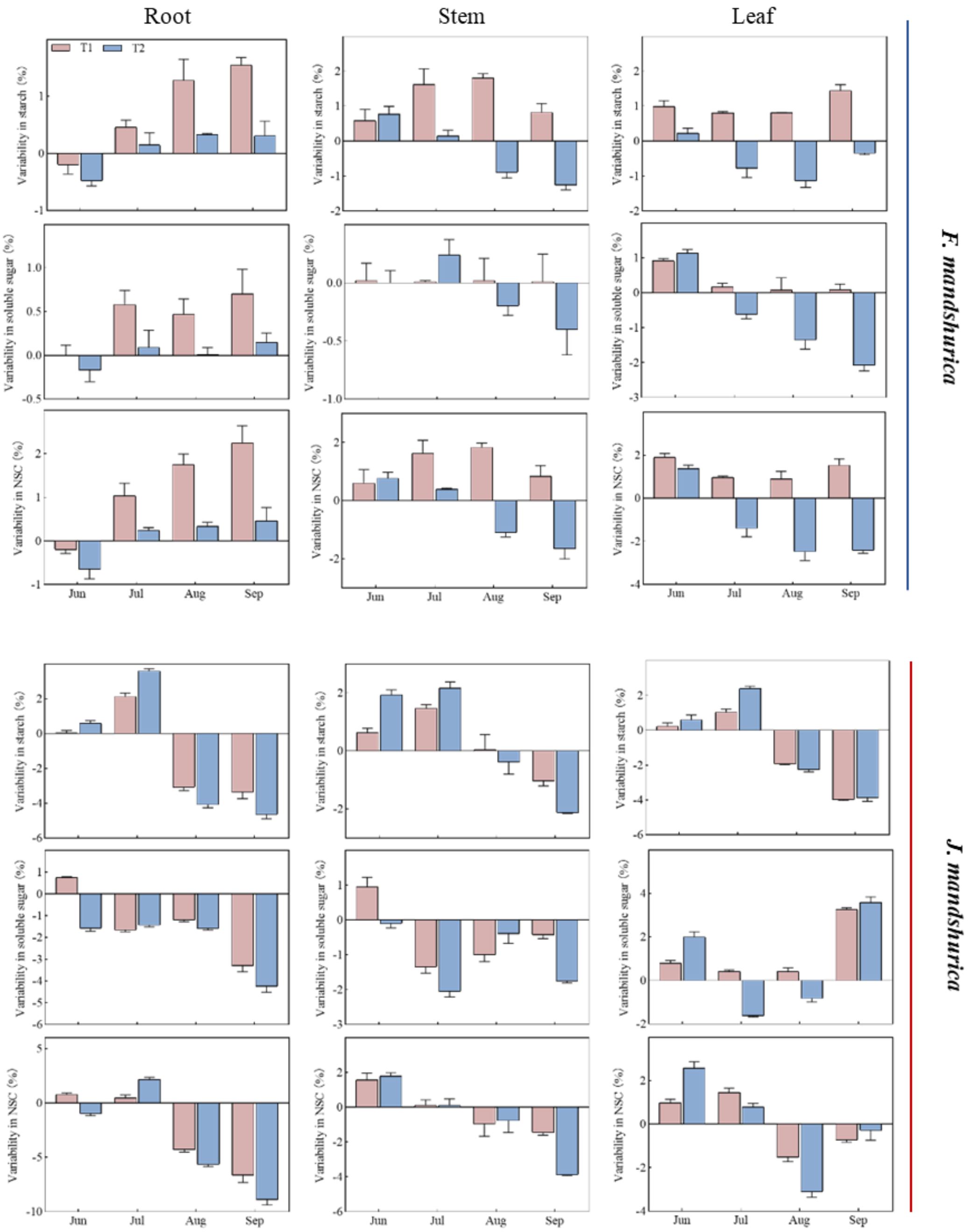

Figure S1 The variability analysis of NSC content of *F. mandschurica* and *J. mandshurica*

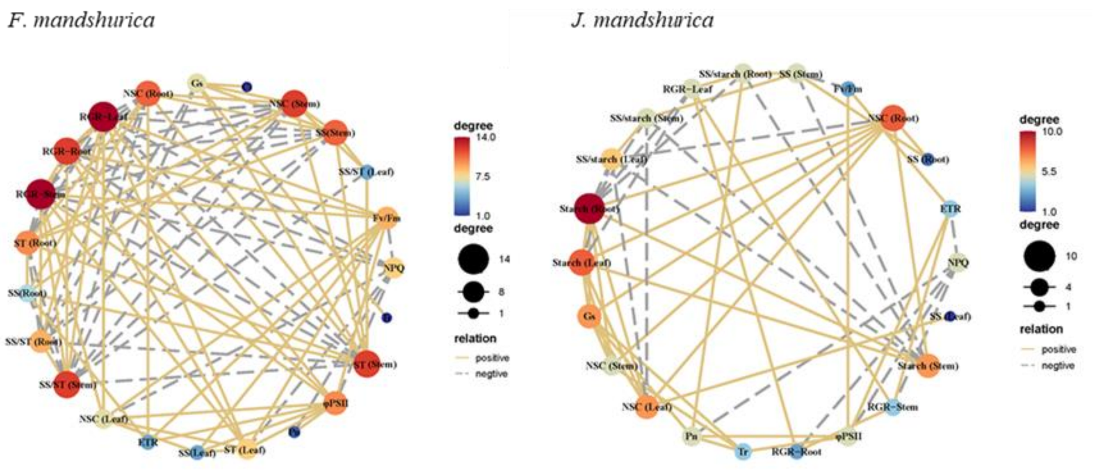

Figure S2 Correlation network analysis of growth parameters of *F. mandschurica* and *J. mandshurica*
