# Supplementary material for: Warming Alters Non-Structural Carbohydrate Dynamics and Source–Sink Regulation in Two Temperate Tree Species
Source: Plants (Basel). 2026 Aug 26;15(17):2598. doi: 10.3390/plants15172598 (PMC13567195; doi:10.3390/plants15172598)
Supplement: Supplementary file 1 [file plants-15-02598-s001.zip › Additional file S2.pdf]

Article

Warming Alters Non-Structural Carbohydrate Dynamics and Source–Sink Regulation in Two Temperate Tree Species

Jingyao Ren [1,†], Jingge Hu [2,†], Zhaoxing Li [1], Lei Jin [3], Kai Huang [4], Yilin Wang [1], Yunze Leng [1], Jiang Liu [1,*,‡] and Xiufen Li [1,5,6,*,‡]

1 College of Agronomy, Shenyang Agricultural University, Shenyang 110866, China
2 Fushun Meteorological Services, Fushun 113006, China
3 Dalian Meteorological Bureau, Dalian 116001, China
4 Key Laboratory of Forest Ecology and Management, Institute of Applied Ecology, Chinese Academy of Sciences, Shenyang 110016, China
5 Qingyuan Forest CERN, National Observation and Research Station, Shenyang 110016, China
6 National Field Observation and Research Station (Liaoning Qingyuan) for Forest Ecosystem, Fushun 113300, China
* Correspondence: snliujiang@syau.edu.cn (J.L.); lixiufen2019@syau.edu.cn (X.L.)
† These authors contributed equally to this work.
‡ These authors co-supervised this work and are co-corresponding authors.

Table S1 Analysis of variance of physiology traits of *F. mandschurica* and *J. mandshurica*

	Variety (A)	Treatment (B)	sampling time (C)	A×B	A×C	B×C	A×B×C
Survival rate	**	**	**	**	**	**	**
RGR-Leaf	**	**	**	**	**	**	**
RGR-Root	*	NS	**	**	**	**	**
RGR-Stem	**	**	**	NS	**	**	**
FvFm	NS	**	**	**	**	**	**
PSII	**	**	**	**	**	**	**

NPQ	**	**	**	**	NS	**	**
ETR	**	**	**	**	**	**	**
Starch (Root)	**	**	**	**	**	**	**
SS (Root)	**	**	**	**	**	**	**
NSC (Root)	**	**	**	**	**	**	**
Starch (Stem)	**	**	**	**	**	**	**
SS (Stem)	**	**	**	**	**	**	**
NSC (Stem)	**	**	**	**	**	**	**
Starch (Leaf)	**	**	**	**	**	**	**
SS (Leaf)	**	**	**	**	**	**	**
NSC (Leaf)	**	**	**	**	**	**	**
Ci	**	**	**	**	**	**	**
GS	**	**	**	**	**	**	**
A	**	**	**	**	**	**	**
E	**	**	**	**	**	**	**
